# Correction: Neuron type-specific proteomics reveals distinct Shank3 proteoforms in iSPNs and dSPNs lead to striatal synaptopathy in *Shank3B*^*–/–*^ mice

**DOI:** 10.1038/s41380-024-02543-3

**Published:** 2024-03-28

**Authors:** Yi-Zhi Wang, Tamara Perez-Rosello, Samuel N. Smukowski, D. James Surmeier, Jeffrey N. Savas

**Affiliations:** 1 Department of Neurology, Feinberg School of Medicine, Northwestern University, Chicago, IL 60611 USA; 2 Department of Neuroscience, Feinberg School of Medicine, Northwestern University, Chicago, IL 60611 USA

**Keywords:** Biological techniques, Neuroscience

Correction to: *Molecular Psychiatry* 10.1038/s41380-024-02493-w, published online 14 March 2024

In this article the title was incorrectly given as ‘Neuron type-specific proteomics reveals distinct Shank3 proteoforms in iPSNs and dSPNs lead to striatal synaptopathy in *Shank3B*^*–/–*^ mice’ but should have been ‘Neuron type-specific proteomics reveals distinct Shank3 proteoforms in iSPNs and dSPNs lead to striatal synaptopathy in *Shank3B*^*–/–*^ mice’.

The original article has been corrected.

