# Supplementary material for: Neuron type-specific proteomics reveals distinct Shank3 proteoforms in iSPNs and dSPNs lead to striatal synaptopathy in Shank3B–/– mice
Source: Mol Psychiatry. 2024 Mar 14;29(8):2372–88. doi: 10.1038/s41380-024-02493-w (PMC11412912; doi:10.1038/s41380-024-02493-w)
Supplement: Supplementary file 1 — Supplementary information [file 41380_2024_2493_MOESM1_ESM.pdf]

Supplementary information

This file included:

Figures and legends for S1-S9

Legends for Tables S1 to S6

Supplemental figures and figure legends

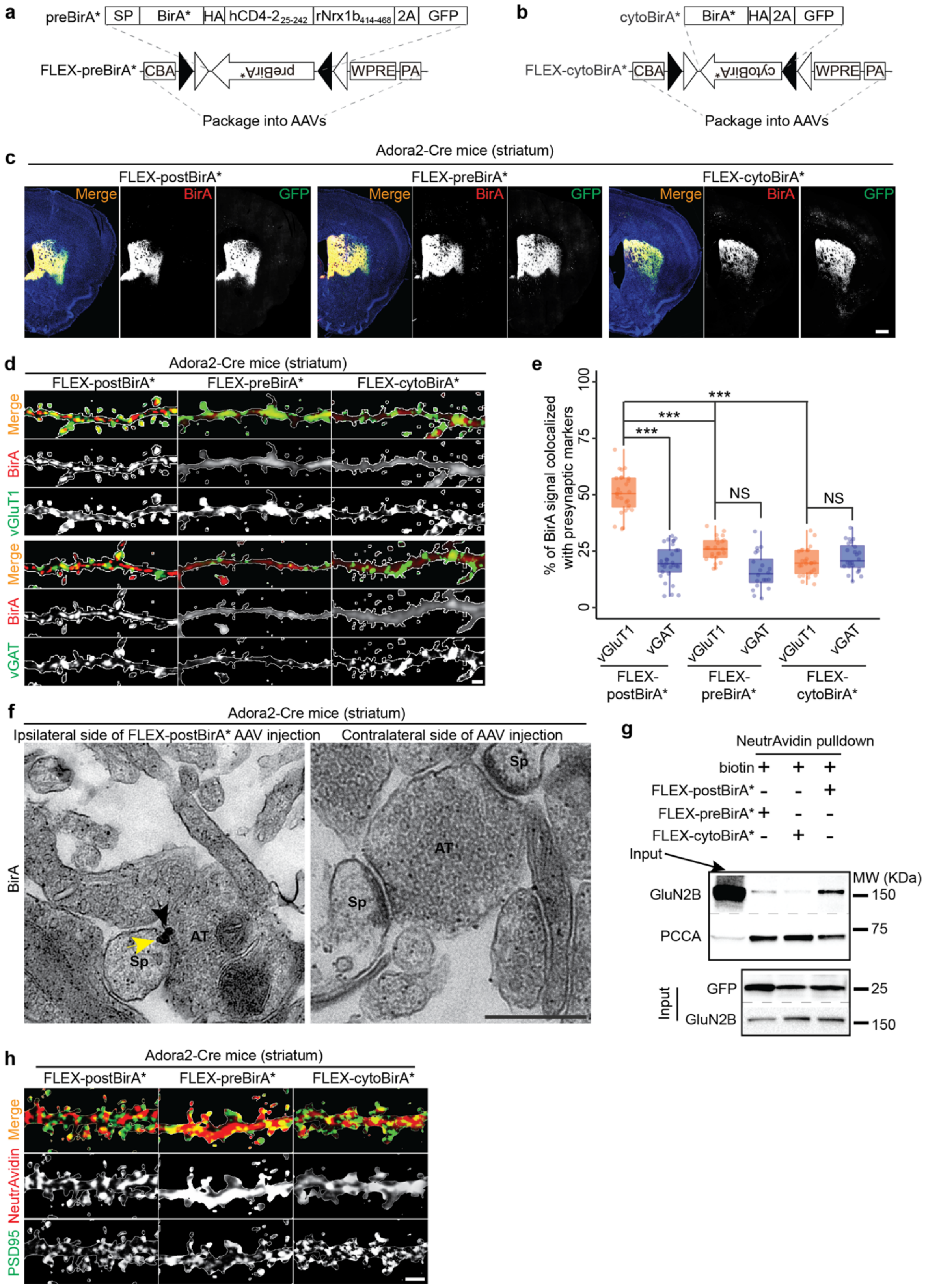

Fig. S1 Confirmation that postBirA* is enriched at postsynaptic sites in striatal iSPNs.

(a) Design of FLEX-preBirA* probe construct. SP, signal peptide, hCD4-2$_{25\text{-}242}$, human CD4-2 amino acid sequence 25-242, 2A, T2A sequence, rNrx1b$_{414\text{-}468}$, rat Nrxn1b amino acid sequence 414-468, CBA, chicken beta actin promotor, WPRE, Woodchuck hepatitis virus posttranscriptional regulatory element, PA, poly-A sequence.

(b) Design of FLEX-cytoBirA* probe.

(c) Expressions of BirA* probes in coronal sections showing expression in dorsal striata of Adora2-cre mice. Scale bar, 0.5 mm.

(d) PostBirA* colocalizes with the excitatory presynaptic marker vGluT1 to a significantly greater degree compared to the inhibitory presynaptic marker vGAT. Neither preBirA* nor cytoBirA* specifically colocalize with vGluT1 or vGAT. Scale bar, 2 μm.

(e) Quantification of **(d)**. n = 4-6 mice, 3-5 brain slices from each mouse. One-tailed Student's t-test, *** p-value < 0.001. NS, no significant.

(f) Immuno-EM micrograph showing two anti-BirA-gold-silver particles, one in the synaptic cleft side (black arrow) and another inside the spine (yellow arrow) from the striatum receiving FLEX-postBirA* AAVs (left). No anti-BirA-gold-silver particles were observed at synapses in the other striatum that did not receive the AAV injection in the same mouse (right). Sp, spine, AT, axonal terminal. Scale bar, 500 nm.

(g) Side-by-side comparison of the affinity purified material from all three probes by WB on the same gel. PCCA is the load control for the purified samples.

(h) NeutrAvidin-Alex568 fluorescent signals in iSPNs are punctate in postBirA* and partially colocalize with PSD95. The another two BirA* probes have contrasting spatial distributions that are not well-colocalized with PSD95. Scale bar, 2 μm.

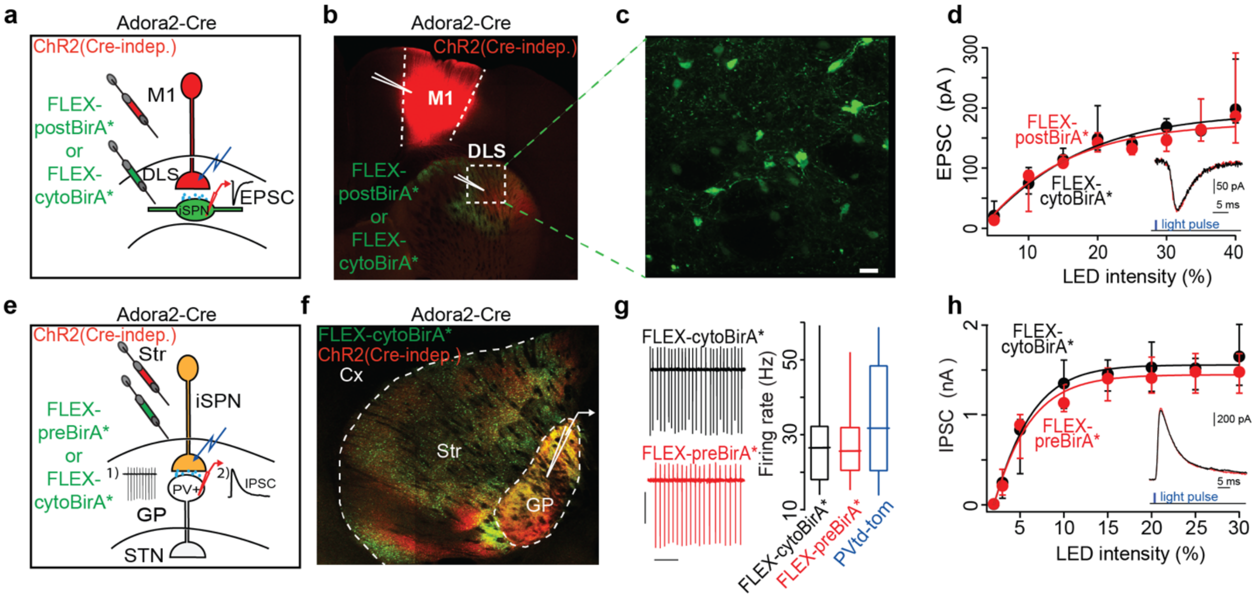

Fig. S2 Unaltered corticostriatal EPSCs and striatopallidal IPSCs in A2a-Cre mice expressing postBirA* or other BirA* probes.
(a) Cartoon showing the experimental design to examine if postBirA* affects corticostriatal EPSCs relative to those expressing cytoBirA*.
(b) Low magnification confocal imaging showing Cre-independent channelrhodopsin-2 (ChR2 Cre-indep.) expression in M1 cortex (mCherry) and probes expression in DLS (GFP).
(c) High magnification confocal imaging showing co-expression of GFP from FLEX-postBirA* AAVs in iSPNs. Scale bar, 15 µm.
(d) Input/output curves for corticostriatal EPSCs at Vm = -80 mV with 15% LED intensity, cytoBirA* = -114 pA (n = 8, 5 mice) vs. postBirA* = -108 pA (n = 9, 6 mice), Bonferroni's multiple comparisons test (p-value > 0.99).
(e) Cartoon showing the experimental design to examine if postBirA* affects striatopallidal IPSCs relative to those expressing cytoBirA*.
(f) Low magnification confocal imaging showing ChR2 expression in striatum (Str) and globus pallidus (GP) (mCherry) and cytoBirA* expression (GFP).
(g) Left, Striatopallidal IPSCs were recorded from presumably parvalbumin positive (PV+) GP neurons at 15-60 Hz. Right, Summary of firing rates (p-value = 0.57, Kruskal-Wallis test), we used historical controls from PV+ neurons recorded in PV tdTomato mice.
(h) Input/output curves of striatopallidal IPSCs at Vm = - 40 mV with 15% LED intensity, ctrl2BirA*: 1455 pA (n = 7,4 mice) vs. preBirA*: 1404 pA (n = 8, 5 mice), Bonferroni's multiple comparisons test, p-value > 0.99). Cx, Cortex, Globus pallidus, GP, Str, striatum, DLS, dorsolateral striatum.

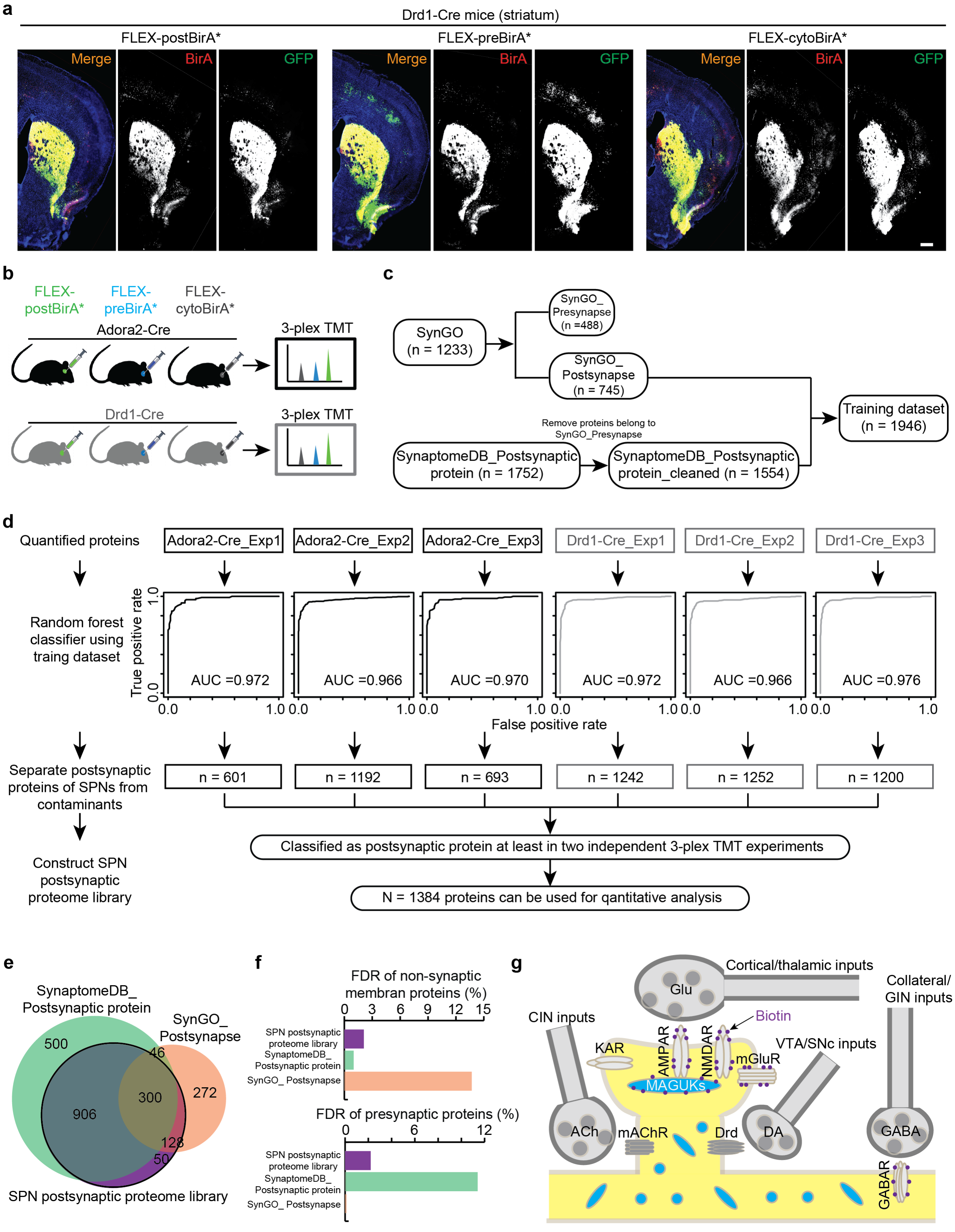

a
Drd1-Cre mice (striatum)
FLEX-postBirA*
FLEX-preBirA*
FLEX-cytoBirA*
Merge
BirA
GFP
b
FLEX-postBirA*
FLEX-preBirA*
FLEX-cytoBirA*
Adora2-Cre
Drd1-Cre
3-plex TMT
c
SynGO (n = 1233)
SynGO_Presynapse (n =488)
SynGO_Postsynapse (n = 745)
Training dataset (n = 1946)
Remove proteins belong to SynGO_Presynapse
SynaptomeDB_Postsynaptic protein (n = 1752)
SynaptomeDB_Postsynaptic protein_cleaned (n = 1554)
d
Quantified proteins
Adora2-Cre_Exp1
Adora2-Cre_Exp2
Adora2-Cre_Exp3
Drd1-Cre_Exp1
Drd1-Cre_Exp2
Drd1-Cre_Exp3
Random forest classifier using traing dataset
True positive rate
False positive rate
AUC =0.972
AUC =0.966
AUC =0.970
AUC =0.972
AUC =0.966
AUC =0.976
Separate postsynaptic proteins of SPNs from contaminants
n = 601
n = 1192
n = 693
n = 1242
n = 1252
n = 1200
Classified as postsynaptic protein at least in two independent 3-plex TMT experiments
Construct SPN postsynaptic proteome library
N = 1384 proteins can be used for qantitative analysis
e
SynaptomeDB_Postsynaptic protein
SynGO_Postsynapse
SPN postsynaptic proteome library
500
46
272
906
300
128
50
f
FDR of non-synaptic membran proteins (%)
FDR of presynaptic proteins (%)
SPN postsynaptic proteome library
SynaptomeDB_Postsynaptic protein
SynGO_Postsynapse
g
Cortical/thalamic inputs
Glu
Collateral/GIN inputs
CIN inputs
Biotin
KAR
AMPAR
NMDAR
mGluR
VTA/SNc inputs
MAGUKs
ACh
mAChR
Drd
DA
GABA
GABAR

Fig. S3 Determine SPN postsynaptic proteome library.
(a) Representative postBirA*, preBirA* and cytoBirA* expression in dorsal striata of Drd1-cre mice. Scale bar, 0.5 mm.
(b) Experimental design for determining high-confidence postsynaptic compartment proteins biotin-tagged by postBirA* in SPNs. Three independent 3-plex TMT-MS experiments, each channel was pooled from three mice for each Cre line. n = 54 total mice.
(c) Generation of training dataset by combination of postsynaptic proteins reported in SynaptomeDB and SynGO databases.
(d) Full set of bioinformatic analyses to determine which proteins can be used for quantitative analysis in further experiments. ROC curves of six 3-plex TMT-MS datasets.
(e) Coverage of SPN postsynaptic proteome library. See Table S1 for the protein list of our library.
(f) For SPN postsynaptic proteome library, the false-discovery rate (FDR) of non-synaptic membrane proteins is 2.1%. The FDR of presynaptic proteins is 2.2%.
(g) Cartoon schematic illustrating selected postsynaptic proteins biotinylated by postBirA* in SPNs. Glu, glutamate, Ach, acetylcholine, DA, dopamine, GIN, GABAergic interneuron, CIN, cholinergic interneuron, VTA, ventral tegmental area, SNc, substantia nigra pars compacta.

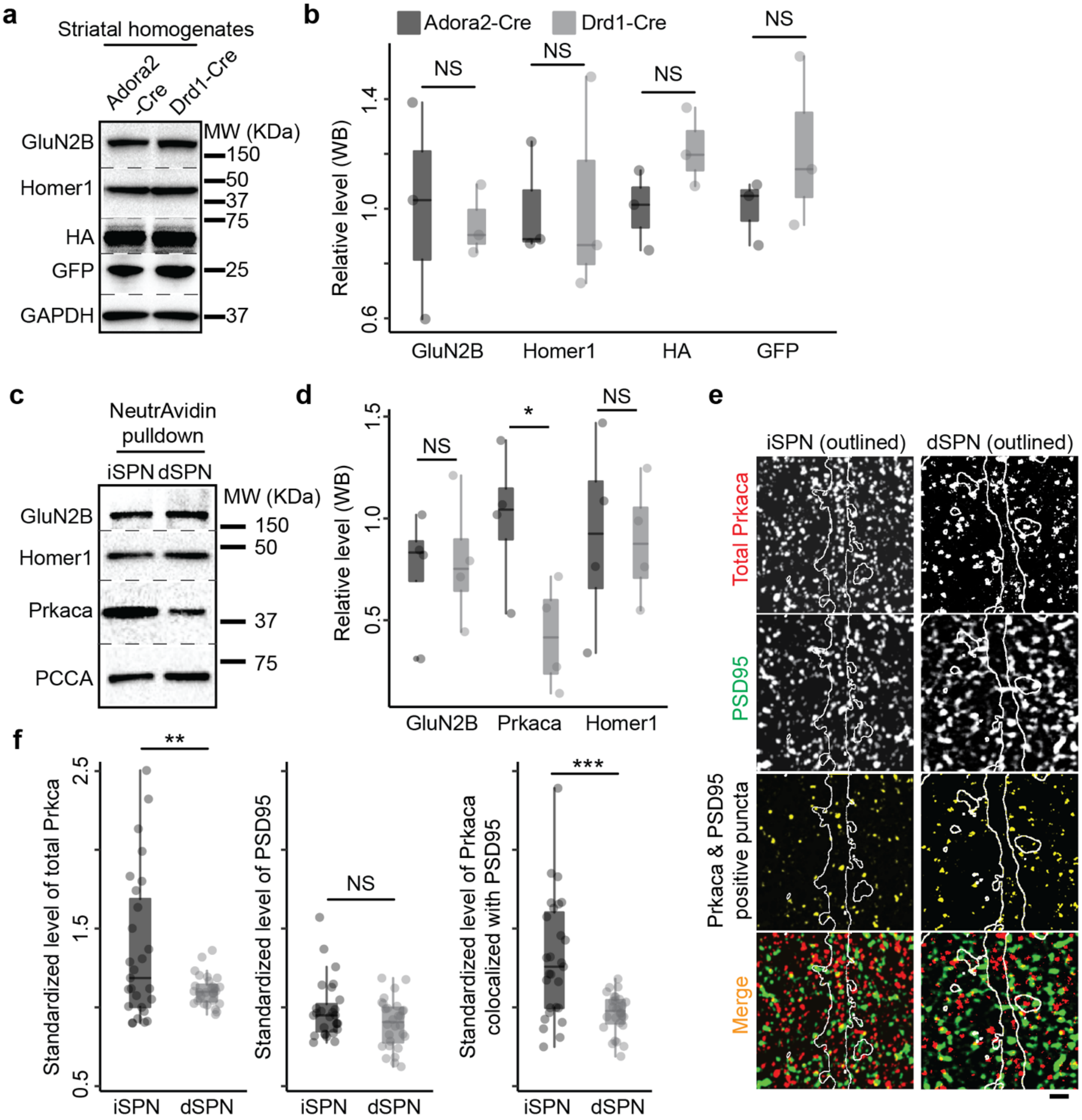

Fig. S4 Validation of TMT-MS data.

(a) The expression levels of postBirA* were similar in Adora2- and Drd1- Cre striata, based on HA and GFP WB.

(b) Quantification of **(a)**, n = 3 mice.

(c) Prkaca level is significantly higher in affinity purified material from Adora2-Cre striata expressing postBirA* compared to Drd1-Cre.

(d) Quantification of **(c)**, protein levels are normalized to PCCA. n = 4 mice per genotype.

(e) PSD95-Prkaca colocalized signals are significantly more abundant in iSPN compared to dSPN dendrites. White outline indicates GFP signal. Scale bar, 1 μm.
(f) Quantification of **(e)**, n = 5 mice, 4-6 brain slices from each mouse.
(b, d & f) One-tailed Student's t-test, * p-value < 0.05, ** p-value < 0.01, *** p-value < 0.001, NS, not significant.

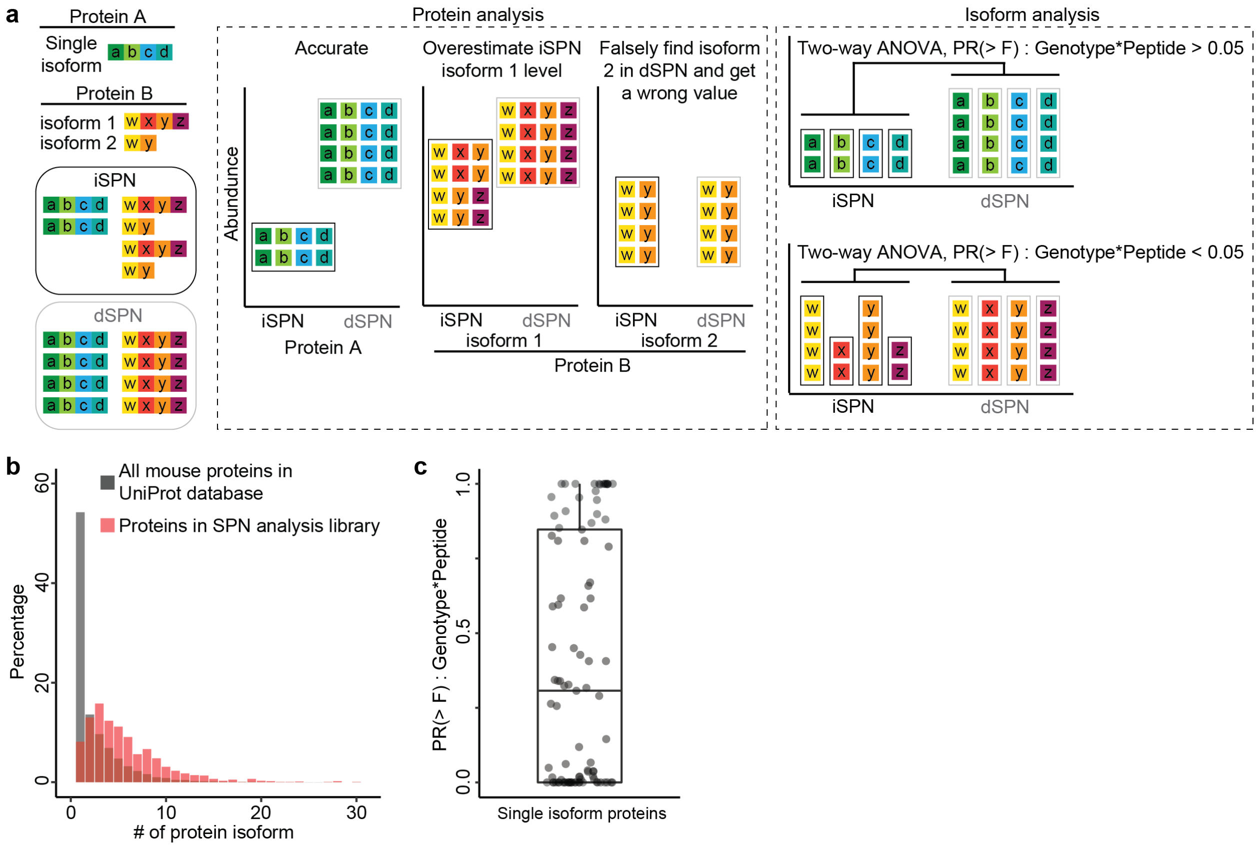

Fig. S5 Logic map of isoform analysis strategy of iSPN and dSPN proteomes by two-way ANOVA.

(a) Comparison of protein analysis and isoform analysis. In protein analysis, the abundance of a protein is calculated based on the relative abundance of all the quantified peptides. This quantification method works very well when the quantified protein only has one isoform (like protein A) or if you consider protein groups. However, it is not well suited to quantify multi-isoform proteins (e.g., protein B). Two-way ANOVA (factors: peptide, genotype) can tell whether the heteroscedastic variances of all quantified peptides are significantly (PR > F): Genotype * peptide < 0.05) related to genotypes. Therefore, isoform analysis can be used to detect divergent expression at the protein isoform level in SPNs. However, isoform analysis cannot be used for quantifications.
(b) Based on UniProt mouse protein database, more than 50% of genes can produce multiple protein isoforms. Interestingly, nearly 90% of genes belonging to our SPN analysis library can produce multiple protein isoforms.

(c) The median of (PR > F): Genotype * peptide values of single isoform proteins is 0.307. The mean is 0.392. The data suggest that the genotype didn't contribute to the variances of quantified peptides of single isoform proteins.

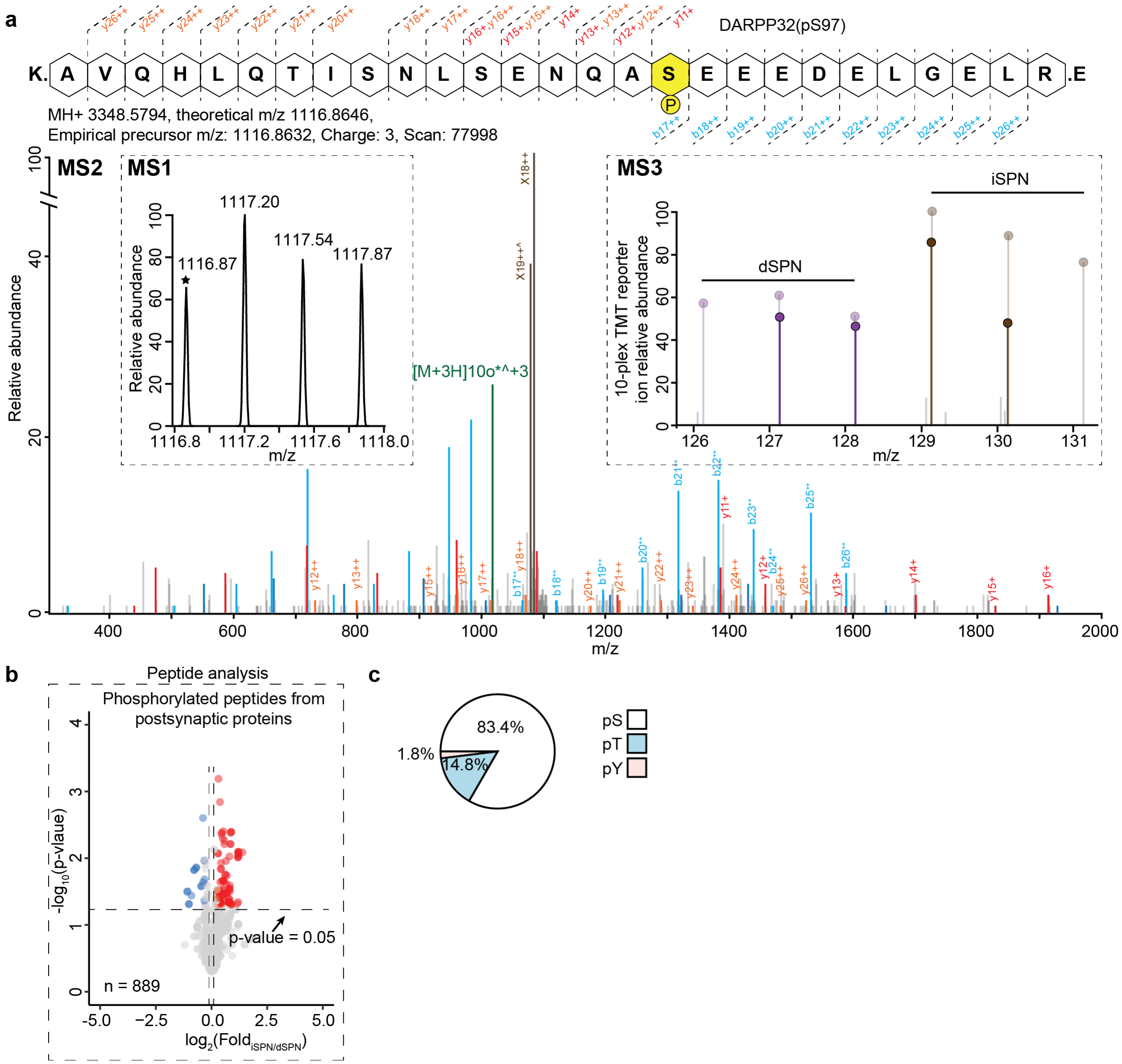

Fig. S6 Differential phosphorylation status of iSPN and dSPN postsynaptic proteins. **(A)** MS spectra of the indicated DARPP32 phosphorylated peptide (pS97) acquired from postBirA* TMT-MS experiment in WT dSPNs and iSPNs. MS1 peak selected for MS2 is indicated with a star. For MS2 spectra assigned b (dark blue +, light blue ++) and y (red +, orange ++) fragment ions are indicated and those containing phosphorylated Serine 97 are labeled. MS3 spectra shows 10-plex TMT reporter ion intensities from dSPNs (purple) and iSPNs (brown).

(B) Peptide analysis showing that the overall level of postsynaptic protein phosphorylation in iSPNs is significantly higher than in dSPNs at the phopshopeptide level. Fisher's combined p-value = 1.84E-89.
(C) Pie charts showed the amino acid compositions of total set of identified phosphorylation sites.

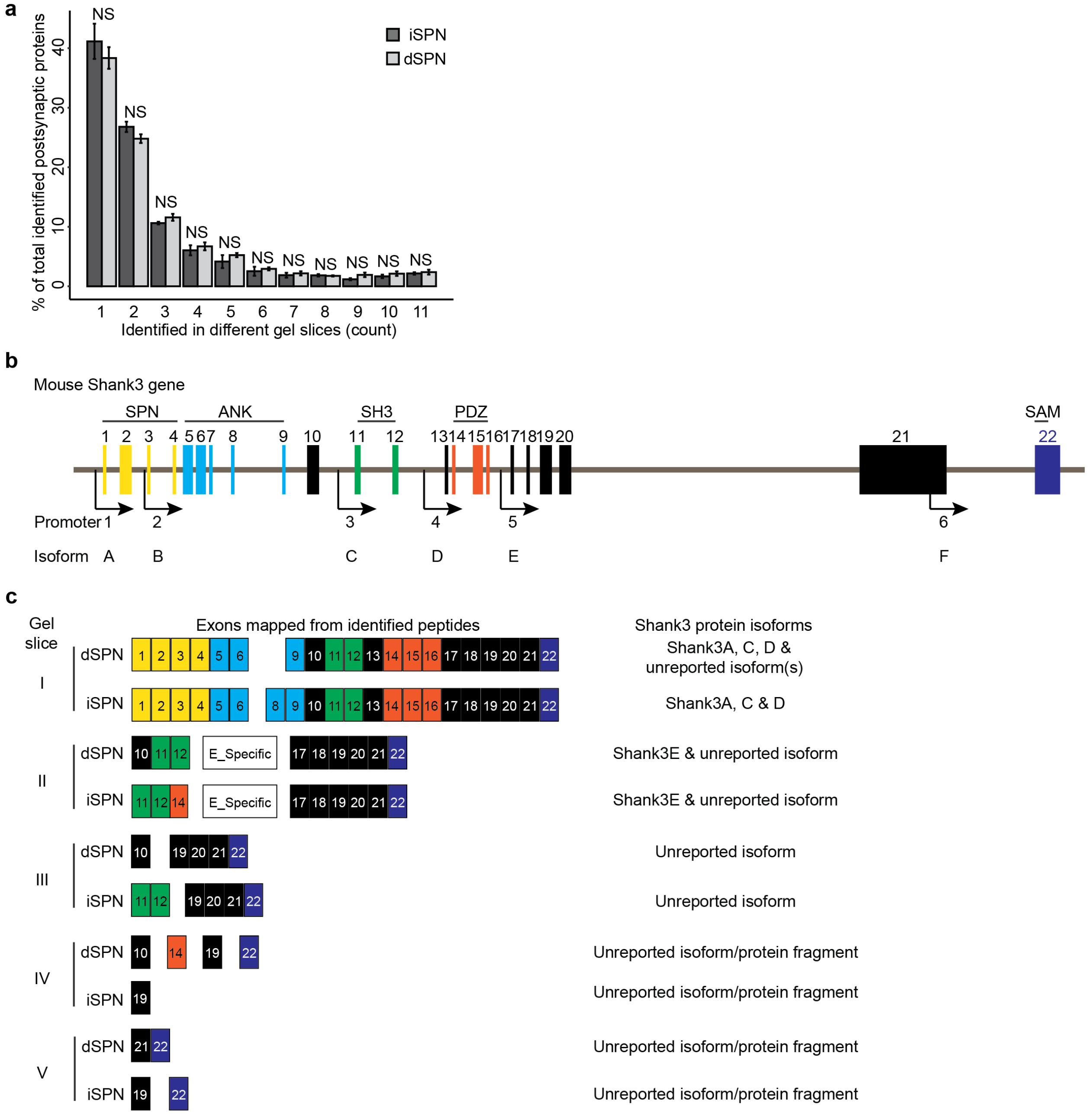

Fig. S7 All Shank3 peptides identified in GeLC-MS^2 experiments mapped to *Shank3* gene exons.
(a) Summary of GeLC-MS2 analysis. Nearly 60% of identified proteins are detected in multiple gel pieces. n = 3 mice for each genotype. Student's t-test, NS, not significant.

(b) Mouse Shank3 gene (Gene ID: 58234) has 22 exons and six known promotors. Alternative usage of promotors directs the expressions of six major Shank3 protein isoforms. ANK, ankyrin repeats domain, SH3, src 3 domain, PRO, proline-rich domain, SAM, sterile α motif domain.
(c) Peptides identified in each gel pieces were mapped to *Shank3* gene exons (NM_021423.4).

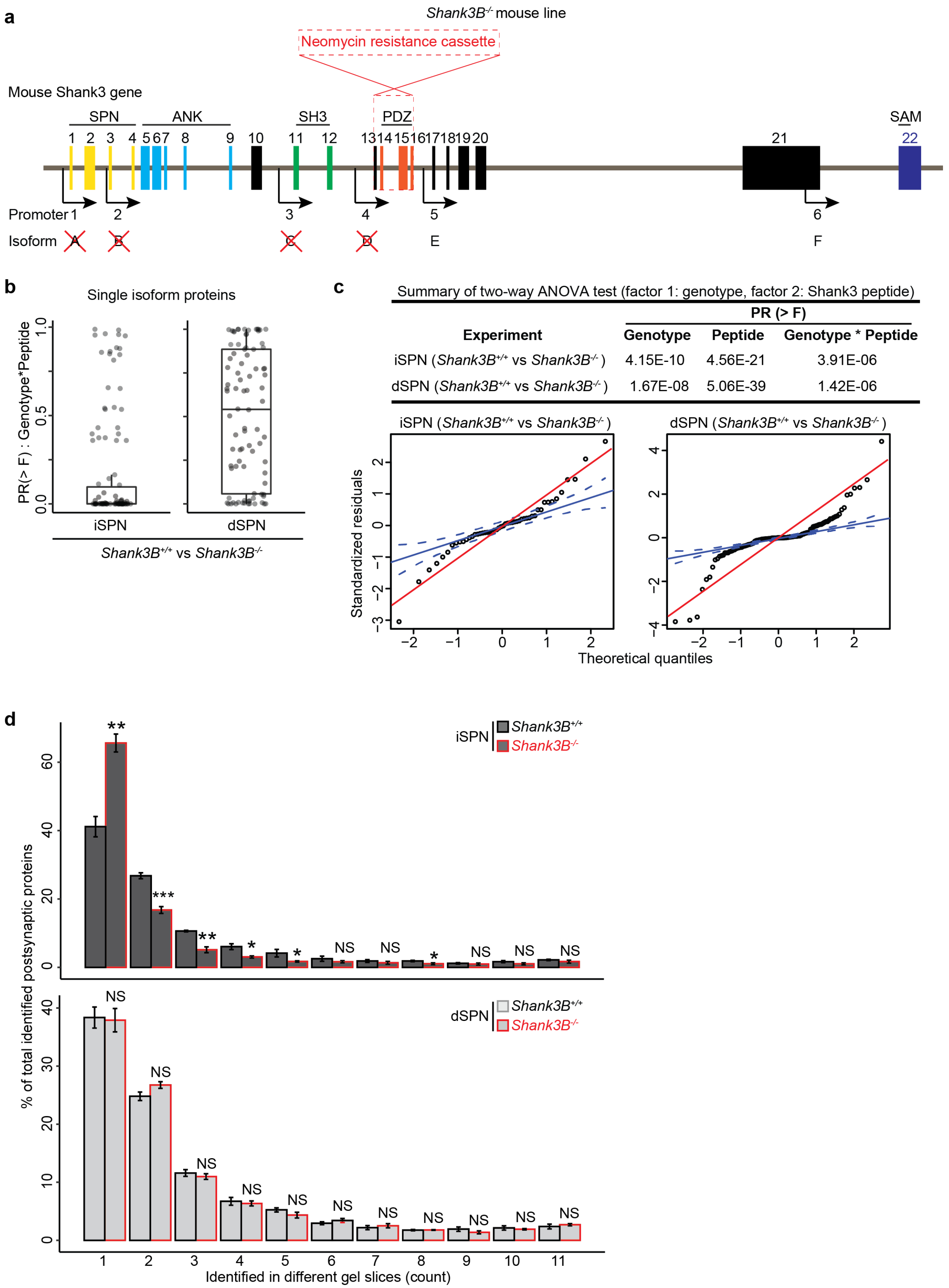
a
Shank3B-/- mouse line
Neomycin resistance cassette
Mouse Shank3 gene
SPN
ANK
SH3
PDZ
SAM
1 2 3 4 5 6 7 8 9 10 11 12 13 14 15 16 17 18 19 20 21 22
Promoter 1 2 3 4 5 6
Isoform A B C D E F
b
Single isoform proteins
PR(> F) : Genotype*Peptide
iSPN
dSPN
Shank3B+/+ vs Shank3B-/-
c
Summary of two-way ANOVA test (factor 1: genotype, factor 2: Shank3 peptide)
PR (> F)
Experiment | Genotype | Peptide | Genotype * Peptide
iSPN (Shank3B+/+ vs Shank3B-/-) | 4.15E-10 | 4.56E-21 | 3.91E-06
dSPN (Shank3B+/+ vs Shank3B-/-) | 1.67E-08 | 5.06E-39 | 1.42E-06
iSPN (Shank3B+/+ vs Shank3B-/-)
dSPN (Shank3B+/+ vs Shank3B-/-)
Standardized residuals
Theoretical quantiles
d
iSPN Shank3B+/+ Shank3B-/-
dSPN Shank3B+/+ Shank3B-/-
% of total identified postsynaptic proteins
Identified in different gel slices (count)
** *** ** * * NS NS * NS NS NS
NS NS NS NS NS NS NS NS NS NS NS

Fig. S8 *Shank3* gene exons 13-16 deletion divergently altered Shank3 protein isoforms in iSPN and dSPN postsynaptic compartments.
(a) In *Shank3B*$^{-/-}$ mouse line, exons 13-16 were replaced with neomycin resistant cassette. It disrupted the expressions of Shank3A, B, C & D.
(b) The (PR > F): Genotype * peptide values of single isoform proteins two 10-plex TMT experiments. For *Shank3B*$^{+/+}$ vs *Shank3B*$^{-/-}$ iSPNs experiment, the median is 0.0007, the mean is 0.142. For *Shank3B*$^{+/+}$ vs *Shank3B*$^{-/-}$ dSPNs, the median is 0.541, the mean is 0.504.
(c) Q-Q plots showing that the variances of Shank3 peptides in *Shank3B*$^{-/-}$ iSPNs and dSPNs are heteroscedastic, which suggests that Shank3 protein isoforms are dissimilar in these two neuron types.
(d) Summary of GeLC-MS2 experiments. Notably, *Shank3* gene exons 13-16 deletion significantly increased the percentage of proteins only detected in single gel piece, which suggests that protein-isoform complexity was reduced in *Shank3B*$^{-/-}$ iSPN postsynaptic compartments. n = 3 mice per genotype. One-tailed Student's t-test, * p-value < 0.05, ** p-value < 0.01, *** p-value < 0.001, NS, not significant.

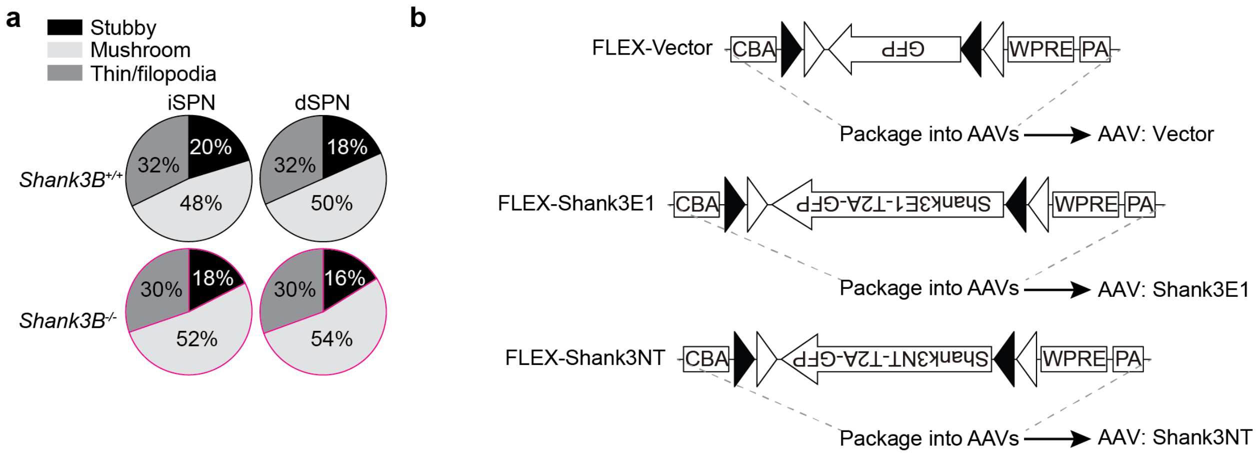

Fig. S9 Constructs for rescue experiments
(a) Proportional distribution of three main spine types in SPN dendrites from mice with the specified genotypes.
(b) Key components of FLEX-Vector and FLEX-Shank3E1 and FLEX-Shank3NT constructs.

Table S1. SPN postsynaptic proteome library
Full list of proteins included in the SPN postsynaptic proteome library.

Table S2. Complete list of quantified postsynaptic proteins in iSPNs vs dSPNs postBirA* TMT-MS analyses
Sheet1: Summary of protein analysis. The indicated intensity values for each biological replicate are calculated from the normalized average of the TMT reporter ion intensities from all of the corresponding peptides spectral matches for each protein. BR = biological replicate (individual mice). One-tailed Student's t-test. SigDn, significantly down-regulated in iSPN postsynaptic compartments (p-value < 0.05, fold$_{dSPN/iSPN}$ > 1.2). SigUp, significantly up-regulated in iSPN

postsynaptic compartments (p-value < 0.05, $fold_{iSPN/dSPN}$ > 1.2). NonSig, no significant difference.
Sheet2: Summary of isoform analysis. Two-way ANOVA test. Similar, (PR > F): Genotype * peptide value > 0.05, dissimilar, (PR > F): Genotype * peptide value < 0.05.
Sheet3: Summary of quantified phosphorylated peptides. The values indicated represent reporter ion intensities for each biological replicate, calculated from the normalized average of the TMT reporter ion intensities from all of the corresponding peptides spectral matches for each phospho-peptides. Student's t-test.

Table S3. List of all Shank3 peptides in GeLC-MS^2 experiments from WT iSPNs and dSPNs.
Sheet 1: All Shank3 peptides identified in gel piece I.
Sheet 2: All Shank3 peptides identified in gel piece II.
Sheet 3: All Shank3 peptides identified in gel piece III.
Sheet 4: All Shank3 peptides identified in gel piece IV.
Sheet 5: All Shank3 peptides identified in gel piece V.
Shank3 peptide sequence, mapped to *Shank3* gene exons, XCorr, ΔCN scores, confidence (Conf%), parts per million (PPM), spectral count (SpecCount) and retention time (RetTime) from Sequest / ProLucid are indicated.

Table S4. Complete list of quantified postsynaptic proteins in *Shank3B*$^{-/-}$ iSPNs and dSPNs compared to WT controls from postBirA* TMT-MS analyses
Sheet 1: Summary of all quantified postsynaptic proteins from postBirA* TMT-MS analysis of iSPNs in *Shank3B*$^{-/-}$ compared to WT control mice. Protein analysis. One-tailed Student's t-test. SigDn, significantly down-regulated in *Shank3B*$^{-/-}$ iSPN postsynaptic compartments (p-value < 0.05, $fold_{Shank3B+/+ /Shank3B-/-}$ > 1.2). SigUp, significantly up-regulated in *Shank3B*$^{-/-}$ iSPN postsynaptic compartments (p-value < 0.05, $fold_{Shank3B-/-/Shank3B+/+}$ > 1.2). NonSig, no significant difference.
Sheet 2: Summary of all quantified postsynaptic proteins from postBirA* TMT-MS analysis of dSPNs in *Shank3B*$^{-/-}$ compared to WT control mice. Protein analysis. Student's t-test. SigDn, significantly down-regulated in *Shank3B*$^{-/-}$ dSPN postsynaptic compartments (p-value < 0.05, $fold_{Shank3B+/+ /Shank3B-/-}$ > 1.2). SigUp, significantly up-regulated in *Shank3B*$^{-/-}$ dSPN postsynaptic compartments (p-value < 0.05, $fold_{Shank3B-/-/Shank3B+/+}$ > 1.2). NonSig, no significant difference.
Sheet 3: Summary of isoform analysis on *Shank3B*$^{+/+}$ vs *Shank3B*$^{-/-}$ iSPN TMT-MS experiment. Two-way ANOVA test. Similar, (PR > F): Genotype * peptide value > 0.05, dissimilar, (PR > F): Genotype * peptide value < 0.05.
Sheet 4: Summary of isoform analysis on *Shank3B*$^{+/+}$ vs *Shank3B*$^{-/-}$ dSPN TMT-MS experiment. Two-way ANOVA test. Similar, (PR > F): Genotype * peptide value > 0.05, dissimilar, (PR > F): Genotype * peptide value < 0.05.

Table S5. List of all quantified Shank3 peptides in *Shank3B*$^{-/-}$ iSPNs and dSPNs compared to WT controls from postBirA* TMT-MS analyses
Sheet 1: All quantified Shank3 peptides *A2a-Cre:: Shank3B*$^{+/+}$ vs *A2a-Cre:: Shank3B*$^{-/-}$ TMT-MS experiment. One-tailed Student's t-test.
Sheet 2: All quantified Shank3 peptides *Drd1-Cre:: Shank3B*$^{+/+}$ vs *A2a-Cre:: Shank3B*$^{-/-}$ TMT-MS experiment. Student's t-test.

Table S6. List of all Shank3 peptides in GeLC-MS^2 experiments from *Shank3B$^{-/-}$* iSPNs and dSPNs.

Sheet 1: All Shank3 peptides identified in gel piece I.
Sheet 2: All Shank3 peptides identified in gel piece II.
Sheet 3: All Shank3 peptides identified in gel piece III.
Sheet 4: All Shank3 peptides identified in gel piece IV.
Shank3 peptide sequence, mapped to *Shank3* gene exons, XCorr, ΔCN scores, confidence (Conf%), parts per million (PPM), spectral count (SpecCount) and retention time (RetTime) from Sequest / ProLucid are indicated.
